# Fabrication of Graphene Polyhedra: Unveiling New Structures, Forms, and Properties

**DOI:** 10.1002/advs.202414108

**Published:** 2025-02-03

**Authors:** Joong Yeon Lim, Seonghwan Kim, Muhammad Toyabur Rahman, Young‐Seong Kim

**Affiliations:** ^1^ Department of Mechanical Robotics and Energy Engineering Dongguk University Jung‐gu Seoul 04620 Republic of Korea; ^2^ Department of Mechanical and Manufacturing Engineering Schulich School of Engineering University of Calgary Calgary AB T2N 1N4 Canada

**Keywords:** alloy, core–shell, graphene polyhedra, metal–organic frameworks, porous carbon

## Abstract

A hybrid nanoporous carbon alloy material is synthesized using a core–shell structure based on metal–organic frameworks, revealing a novel graphene polyhedral form. The presence of carbon and metal as doped cobalt carbides based on morphed graphene within the graphene polyhedra is confirmed through a combination of X‐ray diffraction, X‐ray photoelectron spectroscopy, transmission electron microscopy, and Raman spectroscopy analyses. These novel graphene polyhedra exhibit magnetoelectric coupling properties at room temperature. The magnetic state control is verified using a magnetic probe; the changes in the magnetic state increased with a higher applied bias, and the poling direction of the magnetic phase is reversed based on the scanning direction of the probe. This discovery holds promise for future applications in ultrafast devices and carbon‐based spintronics research.

## Graphical abstract

A unique graphene polyhedral form is developed through the synthesis of a hybrid nanoporous carbon alloy with a core–shell structure derived from metal–organic frameworks. This material demonstrates magnetoelectric coupling at room temperature, verified by magnetic force microscopy, with controllable magnetic states. These findings highlight its potential for advanced applications in ultrafast devices and carbon‐based spintronics.

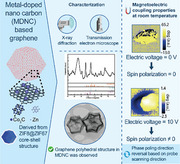

## 1 Introduction

In recent years, extensive research has been conducted on synthesizing nanostructured magnetic semiconductors that exhibit ferromagnetism above room temperature.^[^ ^1^ ^]^ Additionally, the field of carbon‐based spintronic materials has gained considerable research attention, with the focus shifting toward developing nanostructured ferromagnetic carbon materials without the need for synthesizing traditional ferromagnetic substances. Novel structures and forms of graphene and carbon nanotubes (CNTs) have been extensively studied over the past few decades.^[^ ^2^, ^3^, ^4^ ^]^ Research on carbon‐based spintronics using materials like graphene and CNTs has also progressed in various areas. Significant research has focused on spintronics using carbon‐based materials by creating hybrid interfaces, functionalizing surfaces, and incorporating additional materials.^[^ ^5^, ^6^, ^7^, ^8^, ^9^ ^]^

Despite the limitations of carbon‐based magnetic materials, wherein ferromagnetic properties emerge because of residual catalytic materials when ferromagnetic elements are used as catalysts,^[^ ^10^ ^]^ nanostructured carbon materials exhibiting ferromagnetic properties show great potential for spintronics. The magnetic properties of these nanostructured materials hold great promise for applications in spintronics, especially for the development of ultrafast devices, high‐density data storage, and advancements in quantum computing. Covalently bonded carbon nanotubes, which can generate magnetic fields through graphene nanoribbons, are examples of carbon‐based materials that are critical in artificial intelligence applications, where their unique magnetic characteristics play a pivotal role.^[^ ^11^ ^]^

Nanoporous carbon is gaining attention for use in electrodes, adsorption, and catalysis because of its chemical and mechanical stability, excellent electrical conductivity, high surface area, and tunable porosity.^[^ ^12^, ^13^, ^14^, ^15^, ^16^ ^]^ Furthermore, doping of materials can modify material properties and performance.^[^ ^17^, ^18^, ^19^, ^20^ ^]^ The type of metal used for synthesizing carbon can impart specific functionalities to the carbon, with metal‐based carbon materials often exhibiting improved crystallinity and electrical properties.^[^ ^21^, ^22^ ^]^ Leveraging these characteristics, metal‐based carbon materials are being utilized in various applications and have become the focus of significant research across multiple fields.

Recently, extensive research has been conducted on synthesizing carbon materials based on metal–organic frameworks (MOFs), which have controllable pore sizes, high porosities, and large surface areas, enabling the production of various forms for study. Studies have also focused on producing magnetic carbon materials through the carbonization of Co, Fe, and Ni.^[^ ^23^, ^24^ ^]^ The application of these materials by leveraging their unique properties has also been explored for hydrogen storage.^[^ ^25^, ^26^ ^]^

However, nanoporous carbon based on a single MOF structure (e.g., ZIF‐8 and ZIF‐67) has limitations in terms of predictable functionality.^[^ ^27^, ^28^, ^29^ ^]^ Research has focused on heterogeneous hybridization to explore new chemical and physical properties and interfacial functionality to overcome these limitations. This approach has led to increased surface area and pore volume compared with those of single‐component materials, demonstrating superior properties over other porous carbons with hierarchically structured configurations.^[^ ^30^ ^]^ Moreover, the excellent performance of nanoporous carbon has been confirmed through its application in sensor technologies.^[^ ^31^ ^]^ Among these materials, hybrid nanoporous carbon materials have been extensively studied owing to their stable chemical properties and high electrical conductivity, making them suitable for various applications, including energy‐storage electrodes.

Recently, spintronics, which utilizes spin rather than charge, has emerged as a promising technology to reduce power consumption through the electrical control of spin. However, in dilute magnetic semiconductors, which are extensively studied, the availability of materials with Curie temperatures above 300 K is limited. Moreover, challenges such as agglomeration and magnetic instability due to doping concentration, as well as limitations in electrical spin control, remain unresolved. These issues can potentially be addressed using 2D van der Waals materials.^[^ ^32^ ^]^ Nanoporous carbon, on the other hand, offers high material stability and maximizes the interaction of magnetic dopant elements due to its large surface area. Nanographene‐based porous carbon, in particular, exhibits a unique combination of electrical and magnetic properties, making it an exceptionally promising candidate for spintronics applications.^[^ ^33^, ^34^ ^]^

In this study, we explore the formation of metal‐doped nanocarbon (MDNC) materials by adjusting the synthesis conditions to form compounds such as Zn:Co_3_C@Graphene. This study aims to create a material that combines the advantages of nanocarbon structures and metallic ions to develop new forms and structures that exhibit novel electromagnetic properties. To this end, we investigate the structural, magnetic, and electrical properties of the newly formed graphene polyhedra using self‐assembly methods. Through this investigation, we confirm the magnetoelectric coupling properties at room temperature, highlighting the unique features of the new hybrid nanocarbon compound. These characteristics confirm the significant potential of the hybrid nanocarbon compound for various applications, particularly in the field of quantum computing.

## 2 Results and Discussion

**Figure 1:**
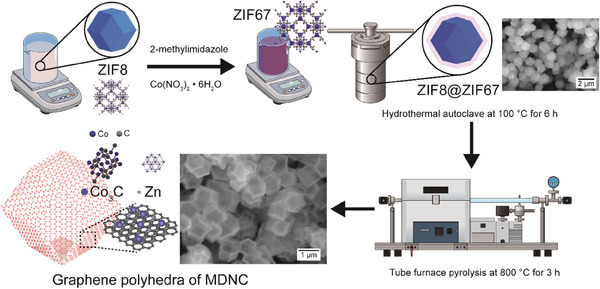
Graphene polyhedra of the MDNC structure synthesis derived from ZIF‐8@ZIF67 and the corresponding SEM images.

Unlike the conventional synthesis method for MDNC, the ratio of cobalt nitrate hexahydrate was reduced to one‐third, and the hydrothermal reaction time was shortened to 6 h in the new synthesis method to successfully produce a new MDNC. The scanning electron microscopy (SEM) images shown in **Figure** **1** indicate that the synthesized material exhibits a highly uniform shape and size; in addition, the final MDNC structure forms graphene polyhedra. First, a ZIF‐8 core with a size of 600–700 nm was synthesized, followed by the formation of a shell using cobalt and 2‐imidazole. The core–shell structure was formed through a hydrothermal process. Unlike previous studies, the hydrothermal time was shortened to form the ZIF‐67 shell, followed by carbonization. Consequently, a uniform core–shell structure and MDNC were successfully obtained^[^ ^31^ ^]^ (Figure 1; Figure S1, Supporting Information).

**Figure 2:**
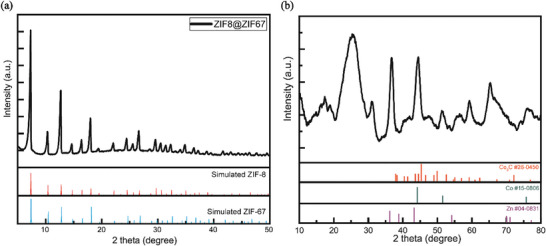
XRD pattern of the a) ZIF‐8@ZIF67 and b) MDNC.

X‐ray diffraction (XRD) analysis was performed to study the structural properties of the new graphene polyhedral structure obtained using the novel MDNC synthesis method, which revealed core–shell and ZIF‐8@ZIF67 and MDNC structures (**Figure** **2**). A typical polycrystalline core–shell structure was formed before heat treatment, as shown in the XRD pattern in Figure 2a; however, after the post‐treatment process, the new structure exhibited XRD peaks at 24.8°, 36.5°, 44.7°, 46.4°, and 51.7°, which were different from previous results^[^ ^30^, ^31^ ^]^ (Figure 2b). When compared to previously reported hybridized nanoporous carbon XRD peaks; instead, it forms a new alloy structure Zn:Co_3_C@graphene. The observed peaks were identified to correspond to morphed graphene structures, specifically Rh‐6 and Rh6‐II graphene, as further validated using high‐resolution transmission electron microscopy (HR‐TEM) analysis.^[^ ^35^, ^36^, ^37^, ^38^ ^]^ Additional peaks reveal the formation of graphitic carbon tilted at 26°,^[^ ^30^ ^]^ as well as the formation of cobalt carbide (Co_3_C) (JCPDS #26‐0450).^[^ ^39^ ^]^ For Zn (JCPDS #04‐0831), peak was observed ≈36°, indicating successful doping, which was further confirmed through X‐ray photoelectron spectroscopy (XPS) analysis. Metallic Co (JCPDS #15‐0806) was detected at 51°.

These findings confirm the formation of a new graphene polyhedral structure in MDNC, which is expected to result in distinct magnetic and electrical properties. This new structure is composed of Zn/Co/C composites, where Co and Zn, along with the frame component C, form an interactive surface both internally and externally. Co reacts with C to form Co_3_C, while Zn is doped in. The doping and alloying processes result in a structure where these elements are integrated with graphene nanosheets, thereby giving rise to a unique graphene‐based polyhedral structure derived from MDNC.

**Figure 3:**
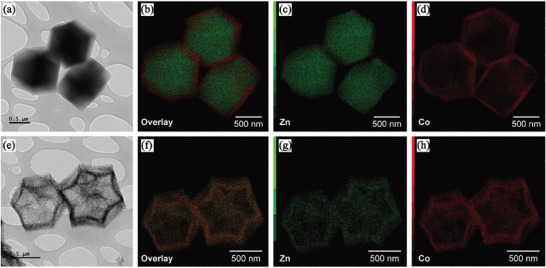
TEM images and EDX mapping of the core–shell and MDNC structures, showing core–shell structure a) topology, b) EDX overlay, c) Zn mapping, d) Co mapping, and MDNC structure e) topology, f) EDX overlay, g) Zn mapping, h) Co mapping.

In the transmission electron microscopy (TEM) images shown in **Figure** **3**, the core–shell structure appears opaque before heat treatment; however, after heat treatment, the MDNC appears in a transparent, thin graphene polyhedral form. Furthermore, energy dispersive X‐ray (EDX) analysis revealed that the outer layer consisted of Co, and the inner core contained Zn before heat treatment; however, after heat treatment, Co and Zn were synthesized in the MDNC composite at the interface. Unlike the previously developed hybridized nano‐porous carbon, this study reduced the Co ratio, shortened the hydrothermal time from 12 to 6 h, and lowered the carbonization temperature to 800 °C. Therefore, the MDNC adopted a different alloy compared to those reported previously.^[^ ^31^ ^]^

The TEM results of the core–shell structure show that the core made of Zn was considerably thicker, whereas that made of Co formed a considerably thinner layer. After heat treatment, the walls of the core–shell structure transformed into graphene walls, with Co and Zn evenly distributed. During the heat treatment process, the outer Co atoms and inner Zn atoms underwent interdiffusion on the carbon surface, leading to the formation of Co_3_C bond structures. Meanwhile, Zn was retained in small amounts due to the high heat treatment temperature and was doped into the stacked graphene, as confirmed by XPS analysis.^[^ ^40^ ^]^

**Figure 4:**
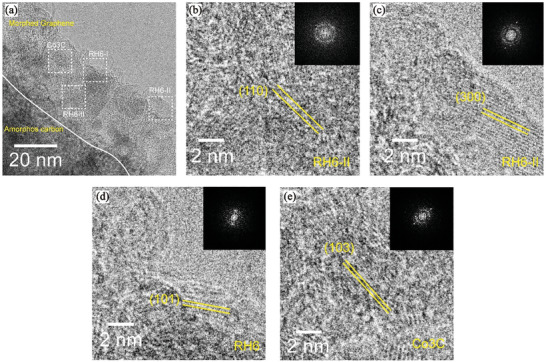
a) TEM image of the MDNC. HR‐TEM images of the MDNC: b, c) RH6‐II morphed graphene, d) RH6 morphed graphene and e) Co_3_C. The insets in (b–e) show the corresponding fast Fourier transform patterns.

To confirm the formation of specific crystalline structures on the surface of MDNC, additional HR‐TEM analysis was performed (**Figure** **4**a). The results revealed that crystalline carbon regions were uniformly formed on the surface, confirming the presence of crystalline facets within the internal surface layer. Specifically, the morphed graphene RH6‐II exhibited (110) planes with a spacing of 3.5 Å and (300) planes with a spacing of 2.0 Å, while RH6 displayed (101) planes with a spacing of 3.0 Å (Figure 4b–d). These observations are consistent with previously reported studies on morphed graphene.^[^ ^35^, ^36^, ^37^, ^38^ ^]^ Additionally, the (103) planes of Co_3_C with a spacing of 1.9 Å were also identified, consistent with JCPDS #26‐0450 (Figure 4e).^[^ ^41^ ^]^ These findings confirm the formation of nanocrystalline carbon along the outer regions of the polyhedral structure.

The synthesized MDNC exhibited morphed graphene walls doped with Co_3_C and Zn. Therefore, the newly formed MDNC based on the graphene polyhedral structure displayed a clear topology of the polyhedral corners of the graphene polyhedral structure after the post‐treatment process; this can be attributed to differences in the synthesis and post‐treatment methods used for the core–shell structure. The EDX analysis also confirmed the formation of Zn:Co_3_C@Graphene within the structure.

**Figure 5:**
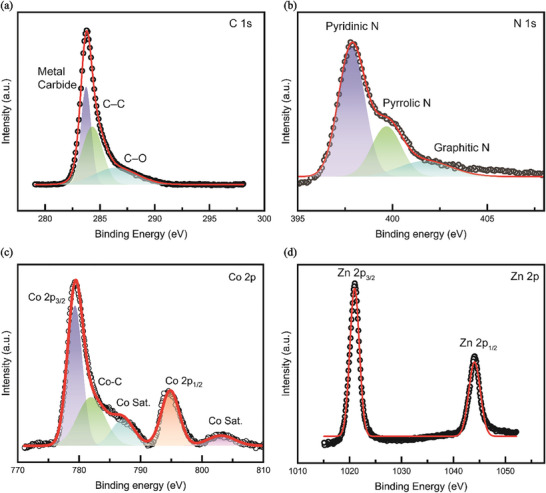
XPS spectra of the MDNC structure, presenting a) C 1s, b) N 1s, c) Co 2p, and d) Zn 2p.

The fitting of the C 1s peaks of the XPS pattern reveals that the metal carbide, C─C, and C─O peaks appear at 283.7, 284.3, and 286.4 eV, respectively. Comparing the results for graphene and graphene oxide indicates that only the C─O peak of graphene oxide is similar, whereas the other peaks differ.^[^ ^42^ ^]^ Compared with the single‐walled CNTs, the C 1s peak shows a general similarity; however, the MDNC peaks are slightly shifted to lower energy values.^[^ ^43^ ^]^ The XPS values of C─C showed a shift toward the metal‐carbide form, indicating bonding with the metals. Such low binding energy values were observed exclusively in metal carbide structures, such as Co_3_C.^[^ ^44^, ^45^, ^46^ ^]^ (**Figure** **5**a; Figure S2, Supporting Information). In addition, N‐doped carbon was analyzed to identify nitrogen functional groups integrated into the carbon lattice^[^ ^47^ ^]^ (Figure 5b). For cobalt, the formation of Co_3_C was confirmed through the Co 2p spectrum, which showed peaks at 779.4 eV corresponding to Co 2p_3/2_ and 782.1 eV attributed to the Co_3_C bond, consistent with previously reported studies (Figure 5c).^[^ ^48^ ^]^ For zinc, the XPS analysis revealed that it remained in a doped state (Figure 5d).^[^ ^49^ ^]^ These findings confirm the formation of Co₃C through the interaction between cobalt and carbon, while zinc remains in its doped state.

**Figure 6:**
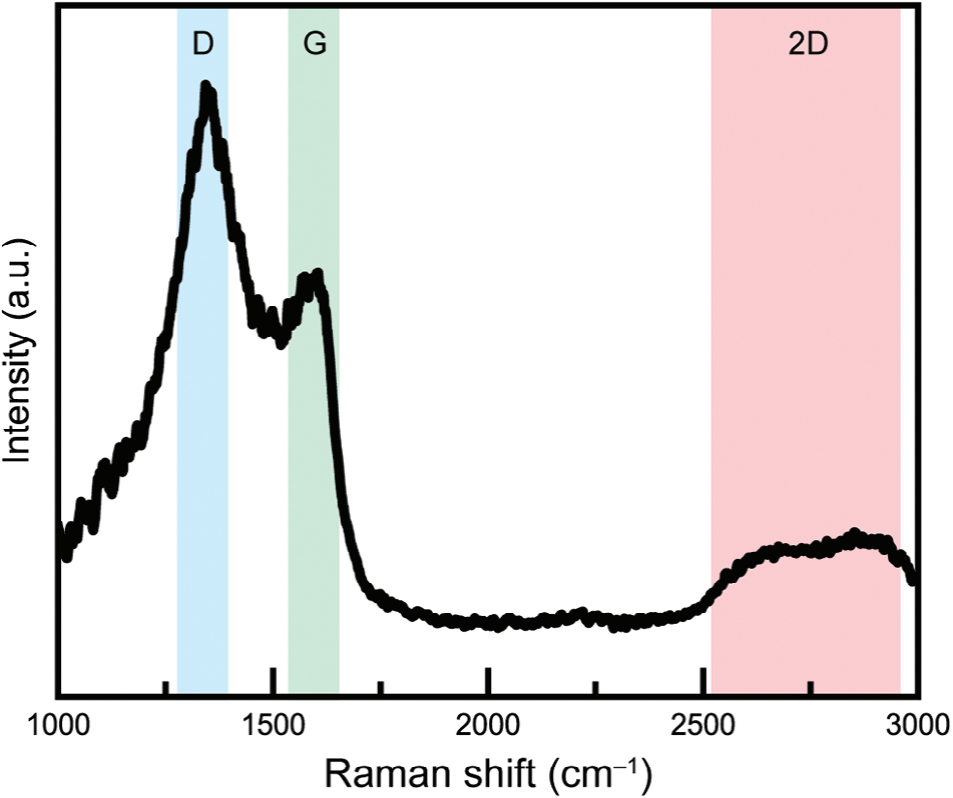
Raman spectrum of the MDNC.

XRD, XPS, SEM, and TEM analysis results confirmed the composition and formation of the new MDNC‐based graphene polyhedral structure. As shown in **Figure** **6**, the Raman spectrum verifies the structural properties. The 2D peak is not strongly visible for graphene oxide (GO); however, the nitrogen‐doped graphene (NGR) peak is pronounced. Unlike the CNTs, where the G peak is more intense, the D peak is stronger in GO, with an I_D_/I_G_ ratio of ≈1. However, the I_D_/I_G_ ratio is ≈2 for NGR, which indicates a significant difference. The NGR exhibits structural similarities to graphite nanoballs^[^ ^50^ ^]^ and nanographene.^[^ ^51^, ^52^ ^]^ The D peak, typically observed at 1355 cm^−^¹ for the A1 g breathing vibration of a sixfold carbon ring, was found to shift to 1344 cm^−^¹ due to the influence of the D peak at 1339.14 cm^−^¹ associated with Co carbide formation.^[^ ^53^ ^]^ Additionally, the G peak appeared near 1600 cm^−^¹, indicating a transition from crystallized graphitic structures to nanocrystalline graphite, thereby confirming the formation of nanometer‐scale clusters.^[^ ^54^ ^]^ The presence of morphed graphene was further supported by the broad 2D peak, which exhibited similar characteristics.^[^ ^37^ ^]^ The analysis of the D, G, and 2D peaks in the Raman spectrum, along with the XPS and TEM results, confirmed that a new structure and set of properties were achieved in the MDNC‐based graphene polyhedra.

**Figure 7:**
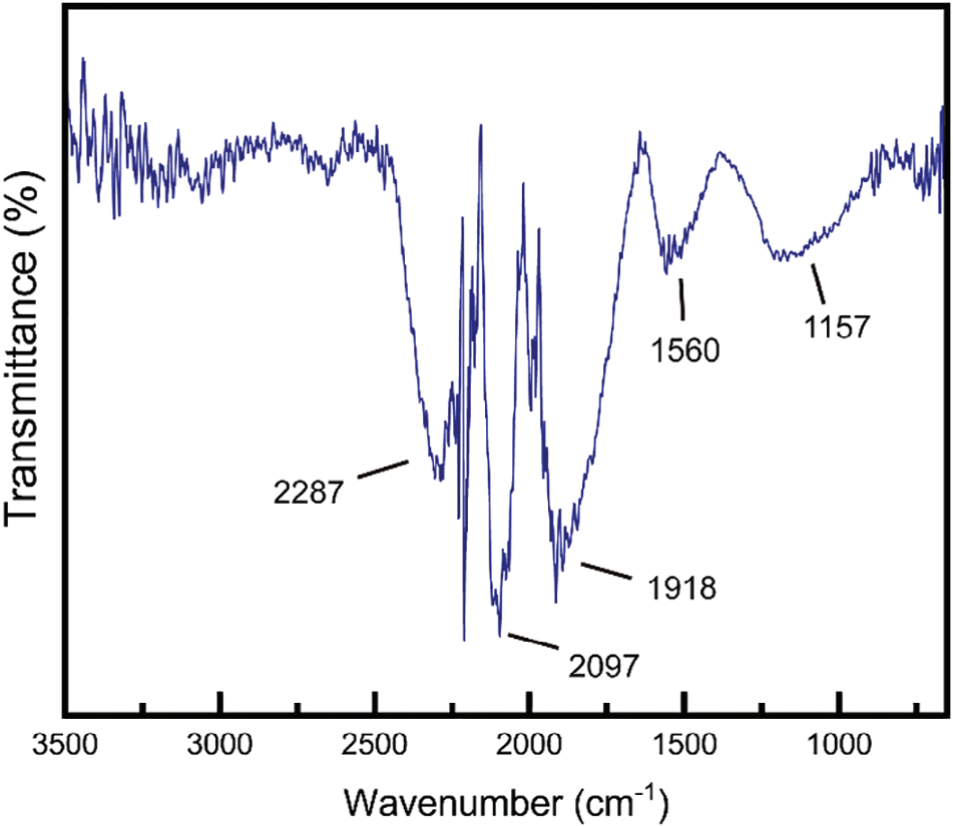
FTIR spectrum of the MDNC.

Fourier‐transform infrared spectroscopy (FTIR) analysis confirmed the distinct formation of Co─C bonding at 1918 cm^−1^.^[^ ^55^, ^56^ ^]^ In addition, various carbon functional groups were identified, including a C─C band at 2097 cm^−1^, a C═C band at 1560 cm^−1^, a C≡C band at 2287 cm^−1^, and a C─O─C band at 1157 cm^−1^ (**Figure** **7**).^[^ ^57^ ^]^

**Figure 8:**
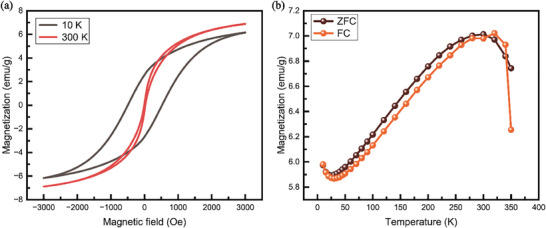
SQUID measurement of the MDNC, showing a) the M‐H curve and b) the M‐T curve. (ZFC: zero field cooling, FC: field cooling).

**Figure** **8**a shows hysteresis results in magnetic moment properties dependent on magnetic field at 10 and 300 K. As shown in Figure 8b, the magnetic properties indicate an antiferromagnetic behavior at room temperature with a Néel temperature of 308 K. The MDNC structure exhibits unique properties unlike those of Co nanoparticles, which typically exhibit superparamagnetic behavior,^[^ ^58^ ^]^ and cobalt oxide (CoO), which has a Néel temperature of 290 K.^[^ ^59^, ^60^, ^61^, ^62^, ^63^, ^64^, ^65^ ^]^ In addition, existing studies on nanoporous carbon materials derived from ZIF‐67 often demonstrate ferromagnetic characteristics at room temperature.^[^ ^29^ ^]^ However, in this study, antiferromagnetic behavior was observed at room temperature, whereas ferromagnetic properties were observed at low temperatures, suggesting that the material is not a composite of single‐phase ZnO or CoO and instead exhibits distinct magnetic characteristics. The reported magnetic properties of CoO, such as its paramagnetic behavior at room temperature, exhibit significant differences compared to the magnetic properties of the present sample. Therefore, the experimental results of this study indicate that the observed magnetic properties can be attributed to Co_3_C.^[^ ^66^ ^]^ These results demonstrate that the newly formed MDNC‐based graphene polyhedra possess unique and well‐defined magnetic properties.

**Figure 9:**
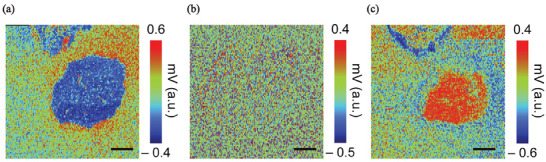
EFM quad (Phase × amplitude) images at a) −10 V, b) 0 V, and c) 10 V (scale bar = 500 nm).

The electrostatic force microscopy (EFM) measurements (**Figure** **9**a–c) clearly reveal charge polarization with an increasing external electric field. No polarization is observed when the external field is 0 V; however, when the field strengthens, distinct polarization is consistently observed across all quadrants of the MDNC‐based graphene polyhedra at ±10 V. This implies that strong polarization is induced by the external electric field. The polarization direction shifts from blue to red as the field changes from −10 to +10 V, indicating a clear change in the polarization angle in response to the direction of the external electric field; this strongly suggests that the polarization angle gradually aligns with the field direction. As shown in Figure 9c , the polarization angle changes by more than 180° in response to variations in the electric field.

**Figure 10:**
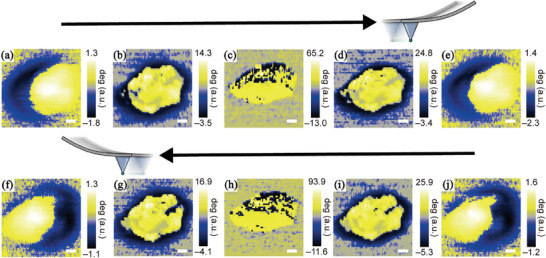
MFM forward images at a) −10 V, b) −3 V, c) 0 V, d) 3 V, and e) 10 V, and backward images at f) −10 V, g) −3 V, h) 0 V, i) 3 V, and j) 10 V (scale bar = 100 nm).

In addition to the charge polarization induced by the external electric field, **Figure** **10**; Figure S3 (Supporting Information) show the magnetic force microscopy (MFM) measurements at room temperature; this demonstrates that spin polarization in the graphene polyhedra is generated through changes in charge induced by the external electric field. The observed spin alignment confirms that the material retains ferromagnetic properties at room temperature. Furthermore, at ±10 V, the spin polarization symmetrically divides into up and down configurations, indicating the formation of distinct spin polarization.(Figure 10a,e,f,j).

No spin polarization is observed at 0 V (Figure 10c,h); however, at 3 V (Figure 10d,i), strong vertical spin polarization becomes visible. The magnetization switching phenomenon becomes apparent in the forward and backward scan directions of the MFM probe and graphene polyhedra under a stronger electric field, which can be attributed to the small internal spin polarizations becoming more sensitive to the scan direction of the probe caused by the strong coupling under the strong external electric field. Thus, a dark/bright contrast of the graphene polyhedra switching left and right indicates that the spin polarization angle gradually changes in accordance with the scan direction.

**Figure 11:**
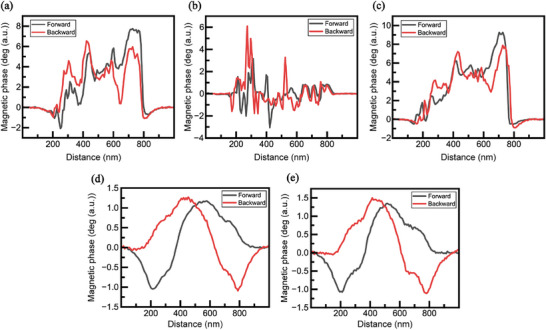
MFM graph at a) −3 V, b) 0 V, c) 3 V, d) −10 V, and e) 10 V.

A slight polarization occurred in the graphene polyhedra at lower voltages; however, a clear and distinct polarization phenomenon was observed at 10 V. **Figure** **11** shows that variations in the magnetic phase caused by the bias and scan direction of the tip are clearly distinguishable when the changes in the images are quantified and compared. Moreover, under high voltage, strong coupling causes the magnetic phase to change symmetrically both in magnitude and direction, depending on the scan direction of the tip.

The degree of polarization, plotted in Figure 11a,c, confirms that the polarization remains in the vertical direction without changing the field direction under a small electric field (±3 V). However, under a large electric field (±10 V), the strong coupling causes the polarization angle to change symmetrically with the scan direction owing to a vortex‐like state formed in the magnetization distribution within the MDNC when the MFM probe moves across the magneton (Figure 11d,e).^[^ ^67^ ^]^ The field from the MFM probe interacts with the magnetization within the MDNC, revealing different magnetization states. This implies that at room temperature, magnetization switching within the nanostructure can be controlled through interaction with the probe based on the scan direction.

To investigate the pore structure of MDNC, Brunauer‐Emmett‐Teller (BET) analysis was conducted, revealing a surface area of 540.5 m^2^ g^−1^, a pore volume of 0.388 cm^3^ g^−1^, and an average pore size of 28.7 Å (Figure S4, Supporting Information).

The MDNC‐based graphene polyhedra exhibit magnetoelectric properties at room temperature. The strength of the external electric field can be adjusted by utilizing the magnetoelectric properties of the nanostructures, which enables fixed spin polarization under low electric fields and diverse polarization changes under strong electric fields depending on the applied direction. Thus, it is suitable for applications such as ultrafast multibit devices for reading and writing in various forms.

## 3 Conclusion

We investigated the formation and properties of a novel MDNC‐based graphene polyhedral structure. Structural analysis confirmed the formation of a novel Zn:Co_3_C@graphene configuration. We identified the unique magnetic properties of the new material and observed that the magnetic characteristics could be controlled using an electric field. The magnetic field around the probe changed depending on the direction of the magnetic probe, and the scan became more sensitive to these changes with an increase in the electric field. Furthermore, at 10 V, the magnetic field not only changed but also switched between positive and negative, depending on the direction of the probe, at room temperature. Therefore, this newly developed synthesis method for graphene polyhedra highlights the potential of this material for room‐temperature spintronic applications and as a promising candidate for ultrafast neuromorphic computing materials.

## 4 Experimental Section

### Materials

The 2‐methylimidazole (C_4_H_6_N_2_), zinc nitrate hexahydrate (Zn(NO_3_)_2_·6H_2_O), and cobalt(II) nitrate hexahydrate (Co(NO_3_)_2_·6H_2_O) used in this study were sourced from Sigma–Aldrich.

### Synthesis of ZIF‐8

First, Zn(NO_3_)_2_ (3.3 g) was dissolved in methanol (45 mL) and 2‐methylimidazole (3.7 g) was dissolved in methanol (90 mL). While stirring the Zn(NO_3_)_2_ solution, the 2‐methylimidazole solution was slowly added and mixed for 2 h. Further, the mixture was incubated at room temperature for 24 h. The resulting product was washed three times with methanol by centrifugation. The washed powder was dried in a vacuum oven for 24 h.

### Synthesis of Core–Shell

The synthesized ZIF‐8 (320 mg) was dissolved in methanol (40 mL) and sonicated for 30 min. Separately, cobalt nitrate hexahydrate (177 mg) and 2‐methylimidazole (3.6 g) were added to methanol (12 mL) and stirred for 20 min. After sonication, the Co and 2‐methylimidazole solutions were sequentially added to the sonicated ZIF‐8 solution and stirred for 5 min. The prepared solution was transferred to a hydrothermal autoclave and allowed to react at 100 °C for 6 h. Once the reaction was complete, the resulting powder was obtained by centrifugation and subsequent drying at 80 °C for 24 h.

### Synthesis of Hybrid Nanoporous Carbon

The core–shell powder was subjected to carbonization in a tube furnace (SH‐FU‐50LTG‐3, SH Scientific) under N_2_ gas conditions at 800 °C for 3 h to obtain the carbonized powder.

### Characterization

XRD was conducted on powder samples using an X'Pert PRO MPD instrument with a Cu‐Kα source at the Korea Basic Science Institute, Seoul Western Center. TEM was performed on the core–shell and MDNC samples were characterized on a field‐emission transmission electron microscope (JEM‐F200, JEOL Ltd.) operating at 200 kV at the National Center for Inter‐University Research Facilities at Seoul National University. Superconducting quantum interference device magnetometry was used for obtaining the magnetic hysteresis and magnetization–temperature curves using a SQUID vibrating‐sample magnetometer (MPMS3, Quantum Design) at the Korea Basic Science Institute, Daejeon Center. XPS was conducted to measure the binding energy of the core–shell and MDNC powder samples using a hybrid X‐ray photoelectron spectrometer system equipped with a UPS and Raman spectroscopy setup (Nexa XPS system, ThermoFisher Scientific) at the Korea Basic Science Institute, Jeonju Center. A monochromatic Al Kα (1486.6 eV) X‐ray source was used. The SEM images were captured using a field‐emission scanning electron microscope (CLARA LMH, TESCAN Brno). Atomic force microscopy (AFM) was performed by drop‐casting the MDNC powder onto a Si wafer and measuring it using the magnetic force microscopy module of the AFM instrument (NX10, Park Systems). The magnetic force was measured under an applied sample bias at the Research Institute of Advanced Materials, Seoul National University. Raman measurements were conducted by drop‐casting the powder onto a Si wafer, followed by drying. The measurements were carried out using an AFM‐RAMAN system (NTEGRA Spectra, NT‐MDT Spectrum Instruments). FTIR analysis utilized a Functional Anal‐ATR‐FTIR spectrometer (IdentifyIR, Smiths Detection), and BET analysis was conducted with a Tristar II 3020 instrument (Micromeritics).

## Conflict of Interest

The authors declare no conflict of interest.

## Supporting information



Supporting Information

## Data Availability

The data that support the findings of this study are available from the corresponding author upon reasonable request.
