# Supplementary material for: Fabrication of Graphene Polyhedra: Unveiling New Structures, Forms, and Properties
Source: Adv Sci (Weinh). 2025 Feb 3;12(15):2414108. doi: 10.1002/advs.202414108 (PMC12005747; doi:10.1002/advs.202414108)
Supplement: Supplementary file 1 — Supporting Information [file ADVS-12-2414108-s001.docx]

Supporting Information

Fabrication of graphene polyhedra: Unveiling new structures, forms, and properties

Joong Yeon Lim, Seonghwan Kim, Muhammad Toyabur Rahman, and Young-Seong Kim*

J. Y. Lim, Y.-S. Kim

Department of Mechanical, Robotics and Energy Engineering, Dongguk University, Jung-gu, Seoul 04620, Republic of Korea
E-mail: [kys4865@dgu.ac.kr](mailto:kys4865@dgu.ac.kr) (Y.-S. Kim)

S. Kim, M. T. Rahman
Department of Mechanical and Manufacturing Engineering, Schulich School of Engineering, University of Calgary, Calgary, AB T2N 1N4, Canada


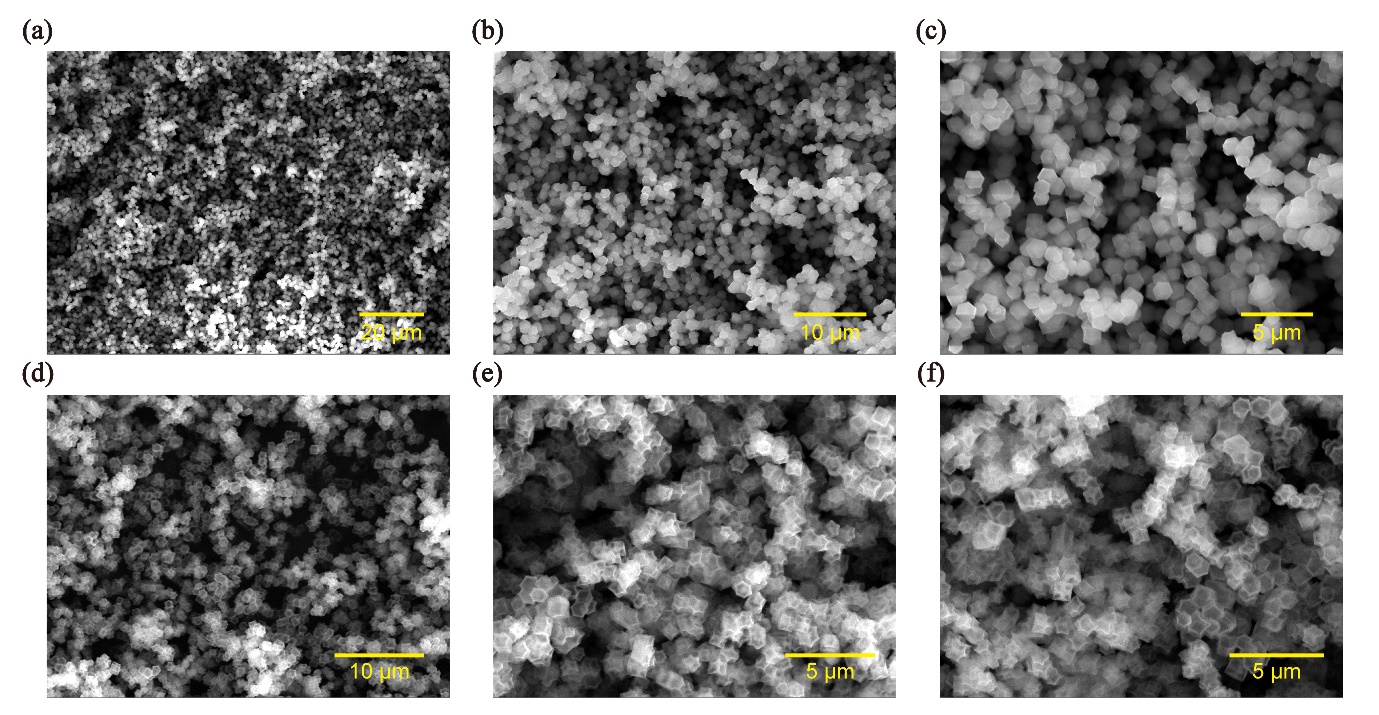


**Figure S1.** SEM images of (a–c) core-shell ZIF-8@ZIF67 and (d–f) MDNC.


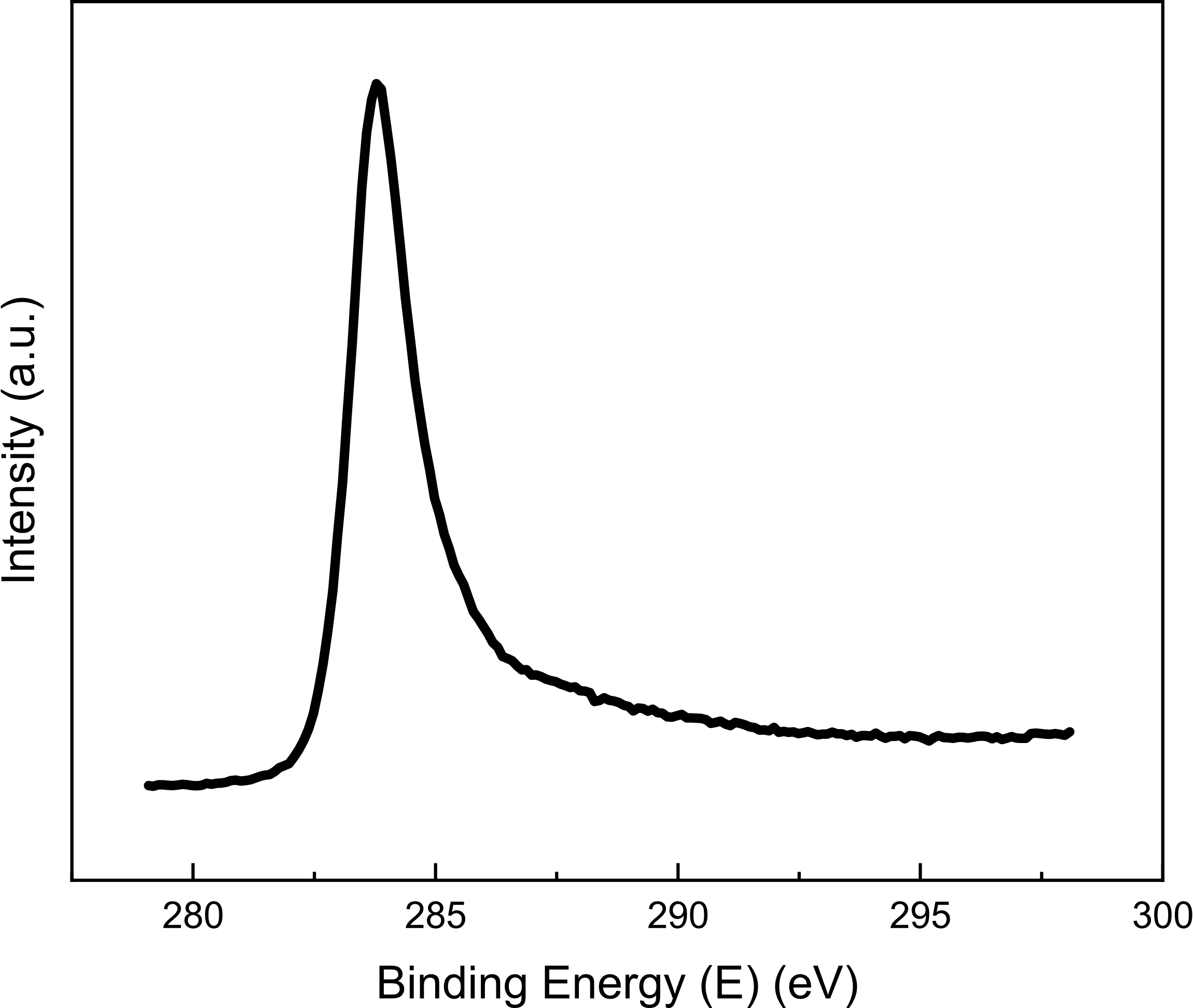


**Figure S2.** XPS spectrum of the MDNC structure showing the C 1s peak.


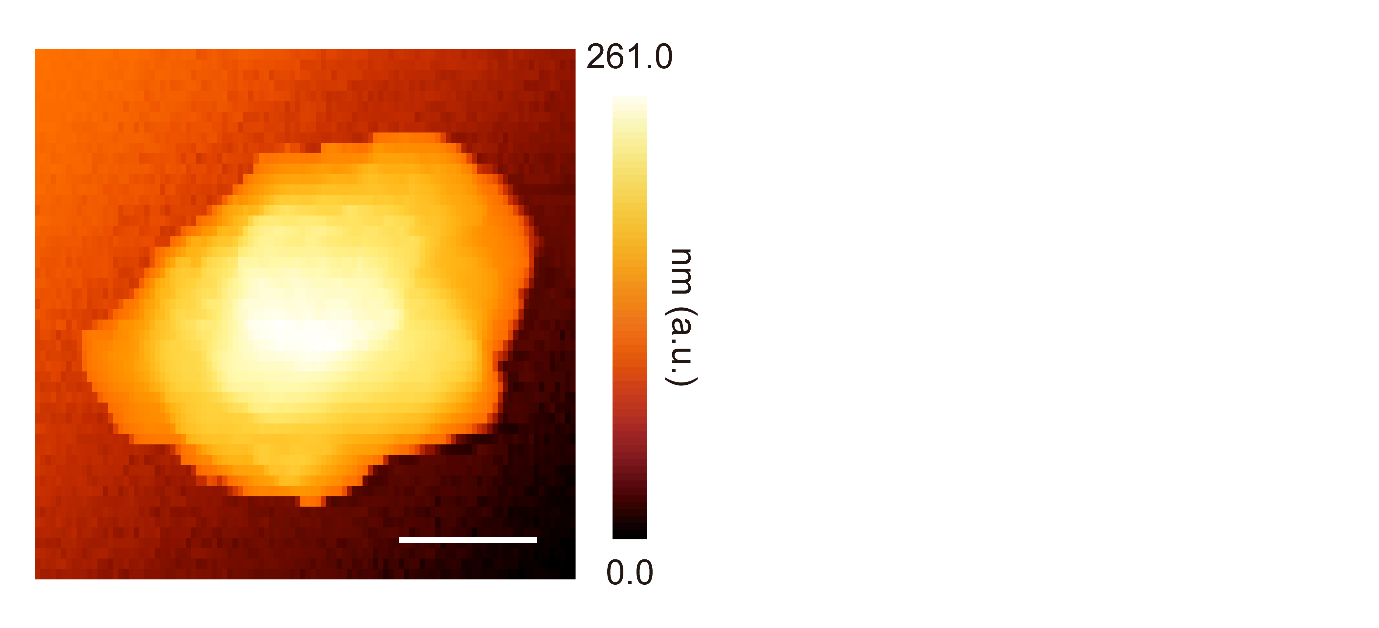


**Figure S3.** MFM topography image ( scale bar = 200 nm).


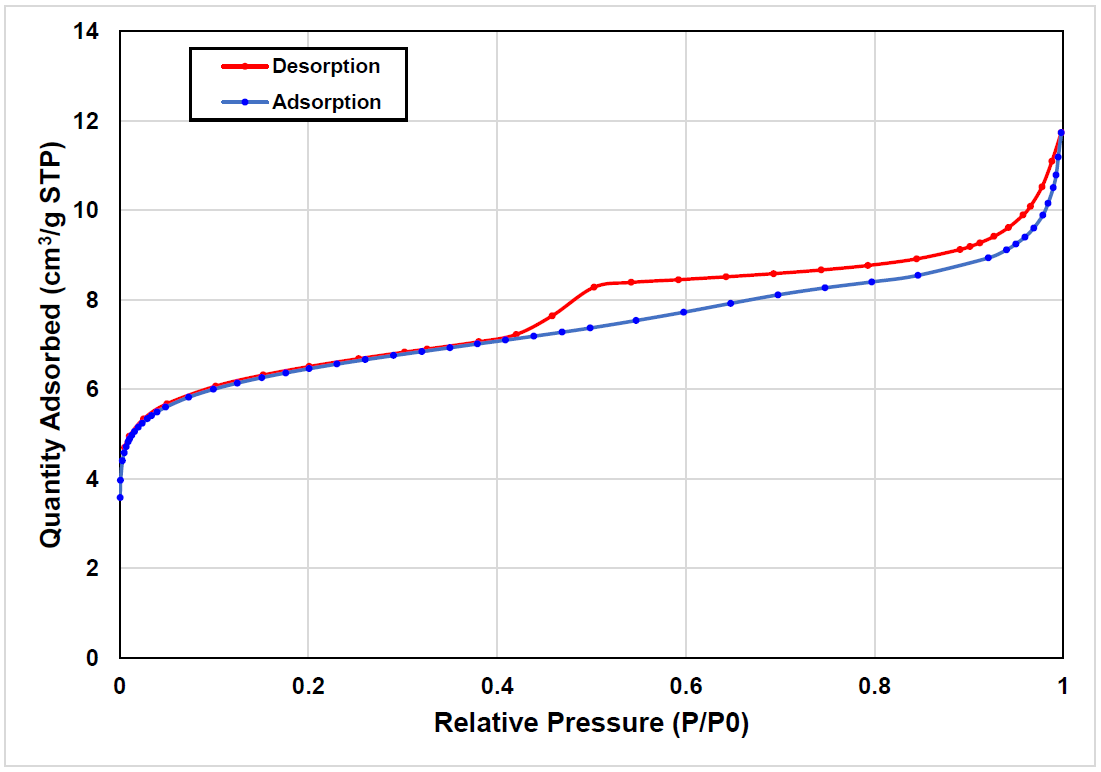
**Figure S4.** Isotherm linear plot of BET surface analysis.
